# The Association of Vitamin D with Non-Melanoma Skin Cancer Risk: An Umbrella Review of Systematic Reviews and Meta-Analyses

**DOI:** 10.3390/medicina59122130

**Published:** 2023-12-07

**Authors:** Konstantinos Seretis, Nikolaos Bounas, Chrissa Sioka

**Affiliations:** 1 Department of Plastic Surgery, Medical School, University of Ioannina, 45110 Ioannina, Greece; drseretis@uoi.gr (K.S.); bounasnikos@gmail.com (N.B.); 2 Department of Nuclear Medicine, Medical School, University of Ioannina, 45110 Ioannina, Greece

**Keywords:** non-melanoma skin cancer, vitamin D, risk, vitamin D receptor, review, VDR polymorphism

## Abstract

*Background and Objectives*: Previous studies revealed the anti-angiogenic, antiproliferative, and anti-inflammatory effects of Vitamin D (VitD) on cancer cells. Although this body of evidence supported the correlation of high VitD levels with reduced incidence rates for various malignancies, contradictory results were reported regarding non-melanoma skin cancer (NMSC). The aim of this overview was to summarize the available evidence from the existing pool of systematic reviews and meta-analyses, focusing on VitD serum status, dietary intake, and VitD receptor (VDR) polymorphisms in correlation to NMSC incidence. *Materials and Methods*: A literature search in electronic databases was conducted from inception to January 2023. The inclusion criteria were systematic reviews and meta-analyses published in peer-reviewed journals, evaluating VitD serum levels, dietary and/or supplementary intake, or VDR gene polymorphisms, and reporting data on NMSC. *Results:* A total of 10 studies were included in the data analysis models. A positive association between VitD serum levels and NMSC is highlighted. However, dietary/supplementation of VitD does not exhibit a likewise strong linkage to NMSC. Despite the contradictory findings, VDR polymorphisms may play a crucial role in the intricate NMSC pathogenesis. *Conclusions:* This umbrella review shows that high VitD levels are associated with increased NMSC incidence, potentially due to its direct correlation with increased sun exposure. Further research on VDR polymorphisms is suggested to explore their true effect size on NMSC risk.

## 1. Introduction

Non-melanoma skin cancer (NMSC) remains the prevailing malignancy in the Caucasian population, displaying a steadily rising yearly rate, with basal cell carcinoma (BCC) and squamous cell carcinoma (SCC) being the most frequent [1,2,3]. Primary NMSC risk factors are considered the cumulative amounts of ultraviolet radiation, as well as the aging population, earlier disease detection, improved registration processes, and climate change [4,5]. Despite the low mortality rates witnessed, these tumors exert a hefty economic burden on health systems and present significant morbidity, specifically in patients with facial tumors, who may require challenging reconstructive operations [6,7].

VitD is a fat-soluble vitamin widely debated over the past decade for its multi-dimensional functionality, currently considered hormonal in nature. Its actions are mediated by its binding to the VitD receptor (VDR), which then translocates to the nucleus to regulate the expression of genes. Apart from its vital contribution to bone and muscle strength, VitD acts as a progenitor of steroidal hormones, and actively modulates the immune system, exerting anti-angiogenic, antiproliferative, and anti-inflammatory effects on cancer cells [8]. The efficiency of these mechanisms is widely supported by the current literature, as several studies correlate high VitD serum levels with reduced incidence rates for various malignancies such as colon, breast, ovarian, and lung cancer [9,10].

The principal source of VitD is cutaneous synthesis through direct sun exposure, while dietary intake contributes to a lesser extent, since only mere quantities have been traced in various foods. However, excessive exposure to ultraviolet radiation induces DNA damage and gene mutations in skin cells, promoting photoaging and skin cancer [11]. Dietary supplements have been introduced to combat the worldwide phenomenon of VitD insufficiency and deficiency, terms corresponding to VitD serum levels <20 ng/mL and between 20–30 ng/mL, respectively, with estimations suggesting that about a billion people display deficient or insufficient vitamin levels [12]. Nonetheless, current data on the association between VitD and NMSC are conflicting [13,14].

The aim of this overview is to summarize the evidence from the existing pool of systematic reviews (SR), and meta-analyses (MA), focusing on VitD serum status, dietary intake, and VDR polymorphisms in correlation to NMSC, synthesize the findings to explore any potential causal relations, and finally investigate contradictive results.

## 2. Materials and Methods

An umbrella review was conducted using a predetermined protocol established according to the Cochrane guide for Overviews of Reviews [15]. The review adhered to the PRIOR (Preferred Reporting Items for Overviews of Systematic Reviews) guidelines (Table S1) [16]. The review protocol was registered at PROSPERO (registration no. CRD42023397779).

### 2.1. Search Strategy

An electronic literature search in the MEDLINE (PubMed), Scopus, and Cochrane Database of Systematic Reviews electronic databases was conducted from inception to January 2023. The string search [“vitamin D”] and [“NMSC”] was applied. The PubMed search strategy can be found in Appendix A. No time and language restrictions were applied. This search was supplemented by a review of reference lists of potentially eligible studies and a manual search of key journals in the field of skin cancer over the last 10 years.

### 2.2. Eligibility of Relevant Studies

The target population was adult patients diagnosed with non-melanoma skin cancer for whom VitD serum levels were recorded. Studies met the following inclusion criteria: (1) systematic review of randomized controlled trials (RCT) or meta-analysis. SRs of both randomized and observational trials were considered eligible for inclusion, only in case they presented a subgroup analysis of RCTs; (2) evaluation of NMSC patients; (3) reported data on VitD serum levels, dietary and/or supplemental intake, or relevant gene polymorphisms; and (4) publication in a peer-reviewed journal. We excluded studies reporting VitD in patients with other cancer types than NMSC, and review articles, duplicate reports, editorials, and correspondences.

### 2.3. Data Collection and Risk of Bias Assessment

Data extraction was conducted independently by two authors using a standardized form. Discrepancies were resolved by consensus. The reviewers extracted the data, including the general study characteristics (including databases searched, primary studies, existence of a pre-registered protocol, reporting according to PRISMA, assessment of risk of bias), population characteristics, and outcomes of interest (estimates of effect and NMSC relative risks assessed by every SR). Data regarding the assessment of publication bias and the heterogeneity of the studies were also recorded.

The quality of the studies was evaluated using the AMSTAR-2 (Assessment of Multiple Systematic Reviews-2) tool [17].

### 2.4. Data Synthesis and Analysis

Narrative synthesis was performed due to the high heterogeneity of the retrieved data. We presented the estimate of the pooled effect sizes of the intervention from included meta-analyses, with their corresponding 95% confidence intervals and *p*-values, with a hazard ratio (HR) for continuous, and an odds ratio (OR) or risk ratio (RR) for dichotomous variables.

To make decisions about the inclusion of overlapping SRs in our overview, we used the evidence-based decision tool, which was developed by Pollock et al. [18]. According to this, we decided to follow the full inclusion scenario, thus including all eligible Cochrane and non-Cochrane reviews, regardless of the extent of overlap, and if they addressed the same research question, as this is the most comprehensive approach [18]. We then assessed the extent of overlapping among the chosen SRs by estimating the overall corrected covered area (CCA) considering chronological structural missingness [19]. We visualized the overlapping SRs with a heatmap using the “ccaR” package in R, version 4.2.3 (R Foundation for Statistical Computing, Vienna, Austria) [20,21].

## 3. Results

**Figure 1:**
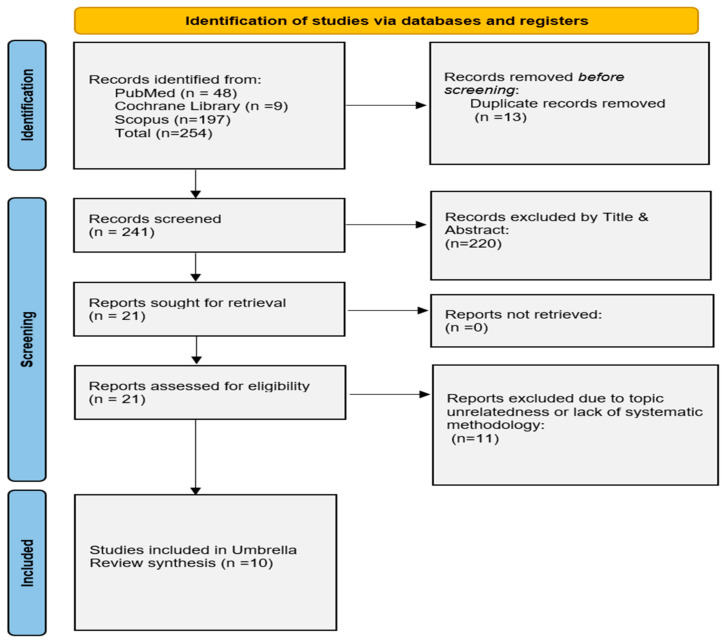
Modified PRISMA flowchart for the overview of systematic reviews.

Our literature search yielded a total of 269 relevant articles. Ten SRs fulfilled our eligibility criteria and were included in the review, with 7 of those conducting meta-analysis of the data [13,14,22,23,24,25,26,27,28,29]. The study selection process is depicted by the flow diagram (Figure 1).

The studies included in our overview were published between 2009 and 2022. Their detailed characteristics are presented on Table 1. The most searched database was Pubmed utilized by all authors, followed by EMBASE and ISI Web of Science. Six of these studies directly investigated the association of Vit D and NMSC, mainly in Europe and the USA, one study investigated VitD and BCC formation while another study focused on several types of cancer including NMSC. The remaining two examined the chemoprotective role of VitD on BCC and SCC, with Sutedja et al. testing its role in patients with confirmed malignancies [27]. Of note, Abdelwahab et al. included 4 studies from Mahamat-Saleh et al. in their review [22].

**Figure 2:**
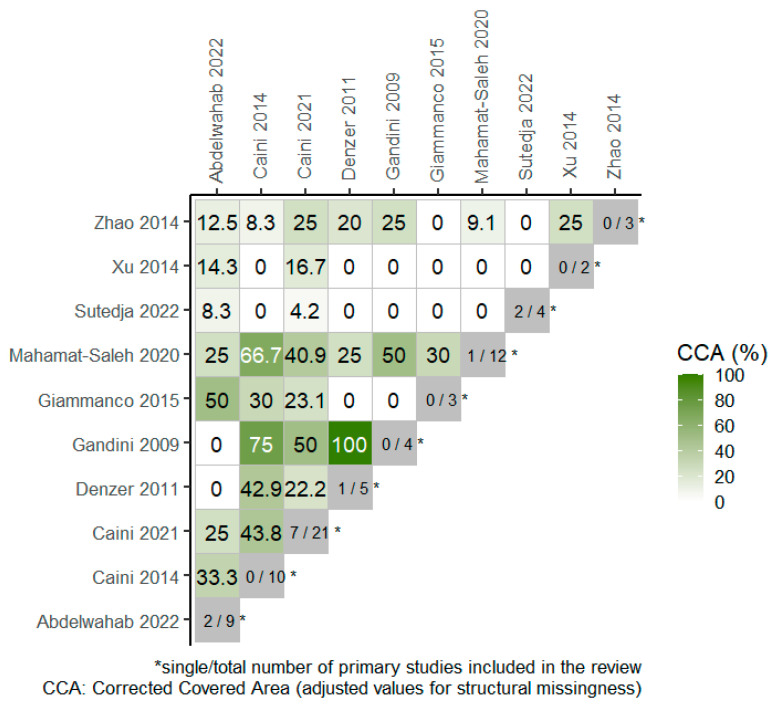
Heatmap for visualization of overlap, adjusted for structural missingness. The degree of overlap of primary studies between pairs of reviews (CCA = 0% represents no overlap of primary studies [white color], CCA = 100% represents complete overlap of primary studies between the SRs [deep green color]) [13,14,22,23,24,25,26,27,28,29].

Overall, 4 RCTs were included in 4 out of the total 10 SRs, included in the overview, as portrayed in the citation matrix (Table S2). The degree of overlap of primary studies across the SRs, based on the overall corrected covered area (CCA) was 15.1% and 23.6% when chronological structural missingness was considered, which indicates high overlap among studies. The heatmap in Figure 2 presents the degree of overlap between pairs of SRs where the CCA values have been adjusted for structural missingness.

Odds ratios (with 95% CI) of the outcomes of interest are analyzed and presented below, as forest plots, along with core information of the overview (Table 2, Table 3 and Table 4).

### 3.1. Vitamin D Serum Levels & NMSC

Table 2 summarizes the study characteristics, regarding the association of VitD serum levels and NMSC. Five SRs, with 3 of those undertaking meta-analyses, reported data on the outcome of interest, incorporating one RCT among the studies. The 3 MAs compared the highest vs. the lowest quantiles of VitD serum level concentration and the formation of NMSC. Two out of three reported a significant positive association for the highest quantile with 1.64 RR ([1.11–2.43], I^2^ = 86%, 249,108 patients) and 1.64 RR [1.02–2.65], I^2^ = 81%, 2317 patients) while the third also marked a trend towards the same direction (1.67 RR [0.61–4.56], I^2^ = 91%, 3899 patients) [13,23,24].

Mahamat-Saleh et al. explored NMSC risk per 30 nmol/L of VitD increments in a dose response meta-analysis [13]. The strongest relative risk for NMSC was observed at a level of approximately 60 nmol/L of 25(OH) D with a weaker association beyond this level. Stratification for BCC and SCC cancer types showed an increased risk for BCC, and a positive trend for the SCC. In their highest vs. lowest quantile analysis, the results were 1.82 RR [1.49–2.21] and 1.80 RR [0.64–5.04]. Caini et al. also performed a stratified quantile analysis for BCC and SCC formation, reporting significant results only for BCC [23]. Furthermore, in their most recent SR, Caini et al. engaged in a linear dose response analysis which showed significant results in 3 of the studies and a trend towards the same direction in two more [24].

Two SRs conducted narrative synthesis of the data, displaying conflicting results. Abdelwahab et al. concluded that 4 studies supported an association between BCC formation and high levels of VitD, whereas the other 4 studies supported an association between BCC formation and low levels of VitD [22]. Giammanco et al. stated that two of the studies included in their review pinpointed a positive association between plasma levels of VitD and NMSC including SCC and BCC, while one study highlighted decreased risk of NMSC in older Caucasian men [26].

### 3.2. Vitamin D Dietary and/or Supplemental Intake

The results of the data extraction on VitD dietary and/or supplement intake and NMSC are presented on Table 3. Five SRs with 2 of those undertaking meta-analyses reported data on the outcome of interest, incorporating two RCTs among the studies.

Mahamat-Saleh et al. concluded that there is a significantly increased risk of BCC with VitD dietary intake (100 UI/day), but also for supplementary intake (100 UI/day), and for total VitD intake [13]. Similar results were reached after a highest vs. lowest quantile analysis for dietary intake, supplemental intake, and for total intake of VitD. However, no significant associations were highlighted in the case of SCC. In their MA, Caini et al. also performed a quantile analysis for VitD dietary intake and discovered a positive trend for NMSC, without achieving statistical significance though [23].

In a recent SR, Caini et al. proceeded in a narrative synthesis of the data they extracted, due to the high heterogeneity observed among the 5 studies they included [24]. Only one study indicated a significant association of increased risk for BCC among those in the highest quantile of intake, while on the other hand, no such outcome was produced for SCC. Gandini et al. discovered a protective effect in the highest quantile for cutaneous melanoma, but when NMSC was incorporated in the analysis no indication of a possible association remained [14]. Sutedja et al. investigated the chemoprotective properties of VitD after administration in patients diagnosed with skin malignancies [27]. Three out of four studies reported a potential protective effect displaying a trend in reducing the incidence rates of NMSC and inducing tumor regression. The fourth study indicated significant tumor regression after combination treatment with VitD and diclofenac.

### 3.3. VDR Polymorphisms and NMSC

The results of the data extraction on VDR polymorphisms and NMSC are presented in Table 4. In total, 5 SRs (4MAs) reported relevant data, incorporating no RCTs.

The Apal polymorphism was studied in relation to NMSC in 3 of the SRs included, giving varying results. Zhao et al. found that genotypes in specific were correlated with significantly increased chances of developing NMSC (Aa vs. AA: 1.72 OR [1.51–2.57], Aa + aa vs. AA: 1.50 OR [1.03–2.17]) [29]. In contrast, Xu et al. demonstrated a significant negative association only for the AA + aa vs. AA genotype with BCC formation (0.59 OR [0.39, 0.91]), as all others were insignificant [28]. On the other hand, Caini et al. presented no significant associations [24].

The Taql polymorphisms were also studied in the previously mentioned 3 SRs. Zhao et al. indicated significant results for 3 genotypes displaying increased chances of developing NMSC (Tt vs. TT: 1.88 OR [1.29–2.74], Tt vs. TT: 2.00 OR [1.22–3.28], Tt + tt vs. TT: 1.92 OR [1.35, 2.73], with Xu et al. supporting these associations by reporting 4 genotypes with an increased likelihood of BCC (tt vs. TT: 2.12 OR [1.21–3.71] I^2^ = 26.4%, Tt vs. TT: 2.14 OR [1.38–3.32] I^2^ = 0%, tt + Tt vs. TT: 2.14 OR [1.43–3.22] I^2^ = 16%, t allele vs. T allele 1.59 OR [1.20–2.11] ] I^2^ = 49.4%) [28,29]. Once again, no significant associations were highlighted by Caini et al. [24].

The Fokl polymorphism was also investigated in 3 SRs. Zhao et al. reported a significant association between the ff vs. FF genotype and NMSC (2.42 OR [1.03–5.68]), with Gandini et al. indicating a positive relationship for Ff and ff vs. wildtype genotypes (1.30 OR [1.03–1.63] I^2^ = 0%) [14,29]. In the SR of Caini et al., one of the 2 studies reporting on the Fokl polymorphism highlighted a significant association for TT (Hom) which greatly increased the chances of BCC (10.14 OR) [24].

The Bsml polymorphism was studied in 4 SRs. In their review, Denzer et al. pinpointed a significant relationship for the BB genotype and SCC (1.51 OR) [25]. Moreover, the interaction between the Bsml polymorphism and high total VitD intake led to an almost 2-fold higher risk of SCC in women with the BB genotype (2.38 OR). Gandini et al. showed positive trends for the Bb and BB genotypes vs. the wild type concerning SCC formation, reporting 1.05 OR [0.76–1.44] and 1.51 OR [1.00–2.28] [14]. On the other hand, Zhao et al. and Caini et al. marked no significant associations for this polymorphism [24,29].

The Cdx2 gene polymorphism has been investigated in 2 SRs, containing no RCTs, indicating no significant associations in relation to NMSC [24,25]. Additionally, one study reported on VitD-binding protein (VDPB) polymorphisms and BCC, without reaching any significant results [24].

### 3.4. Methodological Quality of Included Studies

According to the AMSTAR-2 tool, the overall confidence in the results of the included studies is characterized as low. One study (10%) reported on the existence of a predetermined study protocol [23]. The selection of studies in duplicate was carried out for 2 studies (20%) [23,24]. A higher percentage on duplicate data extraction was reached, since 4 studies (40%) proceeded accordingly [13,23,28,29].

Most of the studies’ literature searches (80%) were considered as “partially comprehensive” [13,14,22,23,24,26,27,29]. A list of excluded primary studies after full-text review, accompanied by the justification for the exclusions, was not provided by any of the included studies. None of the SRs reported on any funding for their included primary studies. All SRs reported potential conflicts of interest in their design, including any funding received. A risk of bias assessment with an appropriate tool was performed in 40% of the SRs [13,22,23,24].

## 4. Discussion

Our overview highlights a positive association between VitD serum levels and NMSC, even though some studies suggest the presence of an inverse relationship. On the other hand, high dietary or supplemental VitD intake does not seem to exhibit a likewise strong linkage to NMSC. Only in one meta-analysis the populations of the highest circulating values displayed increased rates of BCC formation specifically, with low relative risks, however. No such significant differences were reproduced for the SCC subgroups or for NMSC in general among the other SRs reporting on this outcome. Furthermore, despite the contradictory body of the evidence, VDR polymorphisms may partake in the intricate pathogenesis of NMSC. More data are surely mandated to properly establish their influence on skin tumor formation.

VitD has been the focus of intense research because it is regarded as critical, participating in several physiological functions, and thus its blood concentration optimization is suggested. The unique trait of VitD is its synthesis in the body through direct sun exposure. Ultraviolet radiation interacts with a form of cholesterol in the skin, triggering a cascade of reactions eventually resulting in VitD production [30]. Afterwards, by binding to carrier proteins, in particular VDPB, it is transported to the liver and kidneys where it is converted to calcitriol, its active form, also known as 1,25-dihydroxyvitamin D3 [31]. This is the most active form of VitD, which promotes its functions by binding to a single receptor present in almost all tissues of the human body, the VDR, thus regulating gene expression and cellular metabolism [32]. The VDR is a member of the class II of steroidal hormones, showing close relations to the retinoic acid, and thyroid hormone receptor [33]. Of all the genes identified to date, the most powerfully regulated is the CYP24 or 24-hydroxylase enzyme, which is responsible for VitD degradation [33].

Traditionally, VitD is considered to promote calcium and phosphate absorption, maintaining a delicate balance between these two metabolites, and ultimately inducing bone and tooth health. However, VitD also possesses anti-tumor properties, with its ability to impede or delay the growth of specific cancer types [9,10]. This anti-tumor effect is exerted, among others, by regulating cell proliferation. Calcitriol, for example, can prevent the growth of malignant cells by inducing cell cycle arrest, promoting apoptosis, and encouraging the differentiation of both normal and cancerous cells [34,35]. It is also known to stimulate the differentiation of a variety of immature hematopoietic myeloid cells into mature cells [36]. Moreover, it can encourage myeloid leukemia cell lines to differentiate terminally into monocytes/macrophages. Studies conducted on numerous colorectal cancer models have demonstrated the tumor-inhibiting and pro-differentiation effects of calcitriol or its analogs [37]. Additionally, VitD’s anti-cancer properties involve the regulation of androgen and estrogen receptor signaling, while it also plays a crucial role in the modulation of growth factors, oncogenes, and tumor-suppressor gene expression [37]. Inflammation contributes to the development of many cancers, with inflammatory mediators like cytokines, chemokines, and prostaglandins enhancing malignancy potential [38,39]. Recent findings regarding VitD anti-inflammatory properties support its beneficial role in combating carcinogenesis, by regulating key molecular pathways involved in inflammation, such as inhibiting prostaglandin and cytokine synthesis, inhibiting nuclear factor signaling, and suppressing pro-angiogenic factors [37].

In contrast to the evident and well-established protective role of VitD against several malignancies, the results of our overview indicate a positive association between VitD serum levels and NMSC occurrence. Conversely, sun exposure which is the key factor required in the process of VitD production may act as a confounding factor in this association, since data on this aspect is generally lacking [40]. In particular, ultraviolet radiation induces skin carcinogenesis through a complex process involving DNA damage, inflammation, and immune suppression as it penetrates the epidermis and is absorbed by cellular components, specifically DNA [41]. The resulting DNA damage from the production of cyclobutene pyrimidine dimers and reactive oxygen species can cause mutations potentially affecting critical tumor-suppressor genes and proto-oncogenes, that ultimately lead to cancer [41,42]. The implication of oxidative stress in NMSC tumorigenesis has been elucidated by Karampinis et al. and should be considered an added variable of this complex equation [43]. In particular, NMSC patients presented with higher systemic oxidative stress markers than the control group and their oxidative status was influenced by NMSC subtype, VitD sufficiency, and the chronicity of the lesion in question [43]. Critical assessment of this data generates the assumption that VitD levels have to be limited to a specific range and, if possible, its absorption should be maintained through dietary or supplemental intake. Thus, sun exposure should remain reasonable for VitD production and its anti-tumor effects to be achieved, but not be excessive, which can instigate carcinogenesis. Indeed, Mahamat-Saleh et al. support that the strongest relative risk for NMSC peaked at approximately 60 nmol/L, by examining the evidence of non-linear dose response association [13]. The same notion is backed by Abdelwahab et al., concluding that keeping VitD levels between 15 and 30 nmol/L is considered safe, without increasing the risk of tumor progression [22].

The causation effects between sun exposure, NMSC, and VitD presents a complex interplay. Vornicescu et al. discovered that despite comparable outdoor work patterns, individuals with NMSC spent notably more time outdoors than their counterparts [44]. Furthermore, the patient group exhibited lentigines four times more frequently and at least one severe sunburn as opposed to the controls [44]. In general, VitD levels were found to be significantly low, even in the NMSC patients. Similar findings were echoed by Soares et al., whose research highlighted a correlation between elevated VitD levels and increased NMSC rates at diagnosis [45]. One could very well argue though that NMSC cancer patients would avoid further sun exposure after diagnosis which could in turn affect their VitD status. Their study concluded that higher levels of VitD were correlated with increased rates of NMSC [45]. Consequently, measuring serum VitD levels could potentially serve as an indicator of cumulative sun exposure and a predictive marker for NMSC development. Notably, even within a tropical climate, a considerable portion of the NMSC population exhibited insufficient VitD levels, prompting reconsideration of deficiency thresholds. This prompts consideration of whether public disregard for sun exposure risks contributes to inconsistent use of protective sunscreen. Concerning VitD dietary or supplementation intake, most studies display no association. Mahamat-Saleh et al. report, however, a weak, yet positive relationship between increased dietary and/or supplemental VitD intake and BCC formation [13]. Moreover, one study included by Caini et al. showcases similar results [24]. It is extremely hard to construct a concrete explanation of this phenomenon, bearing in mind the mounting evidence of the protective effects that VitD exerts in tumorigenesis. Nonetheless, the association observed was mild and limited only to BCC with data provided solely from observational studies, which are known for their susceptibility to biases. None of these associations were confirmed in the strict context of an RCT. Therefore, it is relatively safe to say that a strong association between VitD intake or supplementation and NMSC is unlikely. In this direction, well-conducted experimental studies should enhance the existing pool of evidence and promote supplementation or intake as a safe way of raising VitD serum levels, thus preserving its manifested benefits.

VitD receptor gene polymorphisms obviously play a significant role in the complex path of NMSC development, according to the results of this overview. The resulting alteration can lead to impaired VitD functionality or even unsettle its own metabolism. For instance, the Fokl polymorphism leads to an entirely different gene product [46]. The f allele, which results in this longer protein, appears to be less effective in the transcription of a VDR, showing its relevance on functional impairment [47]. The Bsml and Taql are 3′-end polymorphisms and display no evidence of a functional effect on VDR activity [48]. Nonetheless, these 3’-end polymorphisms are thought to influence messenger RNA stability and VDR gene transcription regulation [49]. The combined impact of bb, ff, and tt genotypes could potentially result in decreased cellular activity within the VitD system, with some in vitro studies confirming these hypotheses (Bsml/Apal/Taql) [50]. On the other hand, the Cdx2 polymorphism alters the transcriptional activity of the promoter region, as an A allele correlates with increased VDR gene transcription activity [51]. Surely more research is needed on this specific field to properly determine the measures of effect, since relevant studies are relatively few.

Network meta-analyses and umbrella reviews present the most recent advances in the era of evidence-based medicine, enabling the analysis and synthesis of the best data available on a complicated research question, while highlighting questions to be further explored [52,53]. This overview is the first attempt to synthesize all relevant evidence on the effects of VitD on NMSC incidence. Essentially, it provides a methodologically induced summary of the existing information, gathered in a single research paper, contributing to the interpretation of the data, and thus guiding research and clinical decision making. Among the strengths of this overview, is the rigorous methodology implemented, limiting the study risk of bias, and therefore enhancing the presented outcomes of interest. In addition, the utilization of a tool to grade the confidence of the reported results further improved the analysis of the outcomes studied.

Nevertheless, this overview is still subject to limitations. Of note are the methodological quality scores of the included SRs, as evaluated by the relevant but stringent AMSTAR tool, potentially introducing bias. The relatively small number of RCTs contained in the SRs is also worth mentioning. Moreover, the degree of primary study overlap was observed to be remarkable, which is indicative of the reproducibility of outcomes among the SRs.

## 5. Conclusions

In conclusion, despite the cataclysmic positive evidence of the anti-tumor effects of VitD, significant associations have been established between high vitamin serum levels and NMSC incidence. To mitigate this risk while maintaining the health benefits of VitD, alternate sources of the vitamin, such as the dietary or supplementation intake, may be the preferred approaches. This could potentially reduce the well-associated NMSC risk with increased sun exposure. Further research should be conducted on VDR polymorphisms to shed light on our understanding of their true effect size on NMSC genesis.

## Figures and Tables

**Table 1:** Study characteristics.

Author, Year	Databases Searched	Total Studies	Included RCTs	Outcomes Measured	NMSC Cases/Total	Risk of Bias
Abdelwahab, 2022 [22]	Pubmed, Google Scholar, Cochrane Library, Science Direct	10	1	Vit D serum levels & NMSC	28,763/152,488	>70% on each quality assessment tool
Sutedja, 2022 [27]	Pubmed, Scopus, Science Direct	4	3	Vit D supplementation anti-cancer effects on NMSC	955/3003	NR
Caini, 2021 [24]	Pubmed, EMBASE	24	2	Vit D serum levels & NMSC	3899/61,962	Moderate for the observational studies and low for the RCTs
				Vit D Linear dose–response & NMSC	7771/273,991	
				Vit D intake & NMSC	27,299/175,792	
				VDR, VDPB polymorphisms & NMSC	1318/10,284	
Mahamat-Saleh, 2020 [13]	Pubmed	11	0	Vit D serum levels & NMSC	7485/249,108	6 studies with high risk and 5 with moderate risk of bias
				Vit D intake & NMSC	30,981/228,479	
Giammanco, 2015 [26]	Pubmed, ISI Web of Science, Medline, Scopus, Google Scholar	3	0	Vit D serum levels & NMSC	1003/8387	NR
Caini, 2014 [23]	Pubmed, Ovid, Medline, EMBASE, ISI Web of Knowledge	10	1	Vit D serum levels & NMSC	2317/21,964	NR
				Vit D intake & NMSC	7408/156,559	
Xu, 2014 [28]	Pubmed	2	0	Taql polymorphism	332/525	NR
				Apal polymorphism	332/525	
Zhao, 2014 [29]	Pubmed, ISI Web of Knowledge, Medline, EMBASE, Google Scholar	3	0	Fokl polymorphism	918/1984	NR
				Taql polymorphism	332/525	
				Apal polymorphism	332/525	
Denzer, 2011 [25]	Pubmed	2	0	Bsml polymorphism	563/1417	NR
				Cdx2	563/1417	
Gandini, 2009 [14]	Pubmed, ISI Web of Science, EMBASE	5	0	Vit D intake & NMSC	4084/116,953	NR
				Fokl polymorphism	586/1459	
				Bsml polymorphism	586/1459	

**Table 2:** Results on Vit D serum levels & NMSC incidence.

Author, Year	Total Studies/RCTs	Patients	Outcome	Pooled Effect	Heterogeneity (I^2^)
Abdelwahab et al., 2022 [22]	10/1	152,488	Vit D serum levels & BCC formation	4 studies support BCC association with low levels of Vit D and 4 studies support BCC association with high levels of Vit D	-
Caini et al., 2021 [24]	10/0	3899	Highest vs. lowest Vit D concentration & NMSC	1.67 RR [0.61–4.56]	91%
	6/0		Linear dose response	Three studies reported a significant association between increasing Vit D concentration & NMSC, and a trend in the same direction emerged in 2 more.	
Mahamat-Saleh et al., 2020 [13]	8/0	249,108	Highest vs. lowest Vit D concentration & NMSC	1.64 RR [1.11–2.43]	86%
			NMSC risk per 30 nmol/L Vit D increment	1.30 RR [1.13–1.49]	86%
Giammarco et al., 2015 [26]	3/0	13,859	Vit D serum levels & NMSC formation	Positive relationship between plasma levels of Vit D and NMSC including SCC and BCC in 2 studies, while 1 study highlighted decreased risk of NMSC in older Caucasian men	-
Caini et al., 2014 [23]	6/0	2317	Highest vs. lowest Vit D concentration & NMSC	1.64 RR [1.02–2.65]	81%
			BCC	1.82 RR [1.38–2.40]	0%
			SCC	1.68 RR [0.44–6.39]	81%

**Table 3:** Results on Vit D dietary/supplementation & NMSC.

Author, Year	Total Studies/RCTs	Patients	Outcome	Pooled Effect	Heterogeneity (I^2^)
Sutedja, 2022 [27]	4/3	3013	Vit D oral administration & NMSC	Reduced incidence of NMSC in the oral Vit D groups	-
Caini, 2021 [24]	5/2	175,792	Highest vs. lowest quantiles of Vit D intake/supplementation & NMSC	Only 1 study with significant association between highest intake quantile and increased risk for BCC (not for SCC)	-
Mahamat-Saleh, 2020 [13]	5/0	228,479	Vit D dietary intake + suppl. (100 IU/day) & BCC/SCC	1.02 RR [1.00–1.03]/0.99 RR [0.97–1.01]	77.7%/0%
			Highest vs. lowest quantiles of Vit D dietary intake + suppl. & BCC/SCC	1.10 RR [1.05–1.15]/1.02 RR [0.89–1.17]	0%/0%
			Vit D dietary intake (100 IU/day) & BCC/SCC	1.04 RR [1.02–1.06]/1.02 RR [0.97–1.07]	9.8%/0%
			Highest vs. lowest quantiles of Vit D dietary intake & BCC/SCC	1.13 RR [1.08–1.18]/1.14 RR [0.95–1.36]	0%/41.3%
			Vit D suppl. (100 IU/day) & BCC/SCC	1.02 RR [1.00–1.03]/0.98 RR [0.95–1.01]	36.7%/0%
			Highest vs. lowest quantiles of Vit D suppl. & BCC/SCC	1.07 RR [1.03–1.12]/0.95 RR [0.83–1.10]	0%/0%
Caini, 2014 [23]	4/1	7408	Highest vs. lowest quantiles of Vit D dietary intake & NMSC	1.03 RR [0.95–1.13]	0%
Gandini, 2009 [14]	3/0	4084	Highest vs. lowest quantiles of Vit D dietary intake & NMSC	No association	-

**Table 4:** Results on VDR polymorphisms and NMSC.

Author, Year	Total Studies/RCTs	Patients	Outcome	Pooled Effect	Heterogeneity (I^2^)
Caini, 2021 [24]	5/0	2301	VDR gene polymorphisms & NMSC	No associations for Apal, Bsml, Taql, Cdx2. Fokl TT (Hom) significantly increased BCC in one study by 10.14 OR	0% (47% for Hom vs. WT Bsml)
		7983	VDBP polymorphisms & NMSC	One study for VDPB (rs7041, rs4588) with no association for BCC	-
Xu, 2014 [28]	2/0	525	Taql & BCC	tt vs. TT: 2.12 OR [1.21–3.71]	26.4%
				Tt vs. TT:2.14 OR [1.38–3.32]	0%
				tt + Tt vs. TT: 2.14 OR [1.43–3.22]	16.0%
				tt + TT vs. TT: 1.39 OR [0.84–2.31]	0%
				t allele vs. T allele 1.59 OR [1.20–2.11]	49.4%
			Apal & BCC	aa vs. AA + Aa 0.59 OR [0.39, 0.91] while all else were insignificant	0%
Zhao, 2014 [29]	3/0	1984	Fokl & NMSC	ff vs. FF: 2.42 OR [1.03–5.68]	-
			Taql & NMSC	Tt vs. TT: 1.88 OR [1.29–2.74]	
				Tt vs. TT: 2.00 OR [1.22–3.28]	
				Tt + tt vs. TT: 1.92 OR [1.35, 2.73]	
			Apal & NMSC	Aa vs. AA: 1.72 OR [1.51–2.57]	
				aa vs. AA: 1.15 OR [0.72–1.81]	
				Aa + aa vs. AA: 1.50 OR [1.03–2.17]	
			Bsml & NMSC	No association	
Denzer, 2011 [25]	2/0	1417	Bsml & NMSC	BB genotype: 1.51 OR BB genotype + high Vit D intake 2.38 OR in women	-
			Cdx2 & NMSC	No association	
Gandini, 2009 [14]	1/0	563	Fokl & NMSC	Ff and ff vs. wildtype 1.30 OR [1.03–1.63]	0%
			Bsml & NMSC	Bb vs. wild type 1.05 OR [0.76–1.44]	-
				BB vs. wild type 1.51 OR [1.00–2.28]	

## Data Availability

No new data were created or analyzed in this study. Data sharing is not applicable to this article.

## Supplementary Materials

The following supporting information can be downloaded at: https://www.mdpi.com/article/10.3390/medicina59122130/s1, Table S1: PRIOR Checklist; Table S2: Citation matrix of RCTs. Reference [54] is cited in the supplementary materials.

## Appendix A

Pubmed Search String.

(“Vitamin D”[MeSH] OR “Vitamin D”[Title/Abstract] OR “Cholecalciferol”[MeSH] OR “Cholecalciferol”[Title/Abstract] OR “Calcitriol”[MeSH] OR “Calcitriol”[Title/Abstract]) AND (“Skin Neoplasms”[MeSH] OR “Skin Neoplasms”[Title/Abstract] OR “Skin Cancer”[Title/Abstract] OR “Non-Melanoma Skin Cancer”[Title/Abstract] OR “Nonmelanoma Skin Cancer”[Title/Abstract] OR “Basal Cell Carcinoma”[MeSH] OR “Basal Cell Carcinoma”[Title/Abstract] OR “Squamous Cell Carcinoma”[MeSH] OR “Squamous Cell Carcinoma”[Title/Abstract]) AND (“systematic review”[Title/Abstract] OR “meta-analysis”[Title/Abstract])

## References

[1] Demers A.A., Nugent Z., Mihalcioiu C., Wiseman M.C., Kliewer E.V. (2005). Trends of nonmelanoma skin cancer from 1960 through 2000 in a Canadian population. J. Am. Acad. Dermatol..

[2] Staples M.P., Elwood M., Burton R.C., Williams J.L., Marks R., Giles G.G. (2006). Non-melanoma skin cancer in Australia: The 2002 national survey and trends since 1985. Med. J. Aust..

[3] Rogers H.W., Weinstock M.A., Feldman S.R., Coldiron B.M. (2015). Incidence Estimate of Nonmelanoma Skin Cancer (Keratinocyte Carcinomas) in the U.S. Population, 2012. JAMA Dermatol..

[4] Roewert-Huber J., Lange-Asschenfeldt B., Stockfleth E., Kerl H. (2007). Epidemiology and aetiology of basal cell carcinoma. Br. J. Dermatol..

[5] Nan H., Kraft P., Hunter D.J., Han J. (2009). Genetic variants in pigmentation genes, pigmentary phenotypes, and risk of skin cancer in Caucasians. Int. J. Cancer.

[6] Seretis K., Bounas N., Lykoudis E.G. (2023). Repair of a Large Defect Involving the Cheek and Ear. Dermatol. Surg..

[7] Thomaidis V., Seretis K., Fiska A., Tamiolakis D., Karpouzis A., Tsamis I. (2007). The scalping forehead flap in nasal reconstruction: Report of 2 cases. J. Oral Maxillofac. Surg..

[8] Jeon S.M., Shin E.A. (2018). Exploring vitamin D metabolism and function in cancer. Exp. Mol. Med..

[9] Garland C.F., Garland F.C., Gorham E.D., Lipkin M., Newmark H., Mohr S.B., Holick M.F. (2006). The role of vitamin D in cancer prevention. Am. J. Public Health.

[10] Zhang L., Wang S., Che X., Li X. (2015). Vitamin D and lung cancer risk: A comprehensive review and meta-analysis. Cell. Physiol. Biochem..

[11] Narayanan D.L., Saladi R.N., Fox J.L. (2010). Review: Ultraviolet radiation and skin cancer. Int. J. Dermatol..

[12] Holick M.F. (2007). Vitamin D deficiency. N. Engl. J. Med..

[13] Mahamat-Saleh Y., Aune D., Schlesinger S. (2020). 25-Hydroxyvitamin D status, vitamin D intake, and skin cancer risk: A systematic review and dose-response meta-analysis of prospective studies. Sci. Rep..

[14] Gandini S., Raimondi S., Gnagnarella P., Doré J.F., Maisonneuve P., Testori A. (2009). Vitamin D and skin cancer: A meta-analysis. Eur. J. Cancer.

[15] Pollock M., Fernandes R.M., Becker L.A., Pieper D., Hartling L. (2022). Chapter V: Overviews of Reviews. Cochrane Handbook for Systematic Reviews of Interventions.

[16] Bougioukas K.I., Bouras E., Apostolidou-Kiouti F., Kokkali S., Arvanitidou M., Haidich A.B. (2019). Reporting guidelines on how to write a complete and transparent abstract for overviews of systematic reviews of health care interventions. J. Clin. Epidemiol..

[17] Shea B.J., Reeves B.C., Wells G., Thuku M., Hamel C., Moran J., Moher D., Tugwell P., Welch V., Kristjansson E. (2017). AMSTAR 2: A critical appraisal tool for systematic reviews that include randomised or non-randomised studies of healthcare interventions, or both. BMJ.

[18] Pollock M., Fernandes R.M., Newton A.S., Scott S.D., Hartling L. (2019). A decision tool to help researchers make decisions about including systematic reviews in overviews of reviews of healthcare interventions. Syst. Rev..

[19] Pieper D., Antoine S.L., Mathes T., Neugebauer E.A., Eikermann M. (2014). Systematic review finds overlapping reviews were not mentioned in every other overview. J. Clin. Epidemiol..

[20] Bougioukas K.I., Diakonidis T., Mavromanoli A.C., Haidich A.B. (2022). ccaR: A package for assessing primary study overlap across systematic reviews in overviews. Res. Synth. Methods.

[21] R Core Team (2016). R: A Language and Environment for Statistical Computing.

[22] Abdelwahab R., Huang R., Potla S., Bhalla S., Al Qabandi Y., Nandula S.A., Boddepalli C.S., Gutlapalli S.D., Lavu V.K., Mohammed L. (2022). The Relationship between Vitamin D and Basal Cell Carcinoma: A Systematic Review. Cureus.

[23] Caini S., Boniol M., Tosti G., Magi S., Medri M., Stanganelli I., Palli D., Assedi M., Marmol V.D., Gandini S. (2014). Vitamin D and melanoma and non-melanoma skin cancer risk and prognosis: A comprehensive review and meta-analysis. Eur. J. Cancer.

[24] Caini S., Gnagnarella P., Stanganelli I., Bellerba F., Cocorocchio E., Queirolo P., Bendinelli B., Saieva C., Raimondi S., Gandini S. (2021). Vitamin D and the Risk of Non-Melanoma Skin Cancer: A Systematic Literature Review and Meta-Analysis on Behalf of the Italian Melanoma Intergroup. Cancers.

[25] Denzer N., Vogt T., Reichrath J. (2011). Vitamin D receptor (VDR) polymorphisms and skin cancer: A systematic review. Dermato-Endocrinology.

[26] Giammanco M., Di Majo D., La Guardia M., Aiello S., Crescimannno M., Flandina C., Tumminello F.M., Leto G. (2015). Vitamin D in cancer chemoprevention. Pharm. Biol..

[27] Sutedja E.K., Arianto T.R., Lesmana R., Suwarsa O., Setiabudiawan B. (2022). The Chemoprotective Role of Vitamin D in Skin Cancer: A Systematic Review. Cancer Manag. Res..

[28] Xu Y., He B., Pan Y., Deng Q., Sun H., Li R., Gao T., Song G., Wang S. (2014). Systematic review and meta-analysis on vitamin D receptor polymorphisms and cancer risk. Tumour Biol..

[29] Zhao X.Z., Yang B.H., Yu G.H., Liu S.Z., Yuan Z.Y. (2014). Polymorphisms in the vitamin D receptor (VDR) genes and skin cancer risk in European population: A meta-analysis. Arch. Dermatol. Res..

[30] Webb A.R. (2006). Who, what, where and when-influences on cutaneous vitamin D synthesis. Prog. Biophys. Mol. Biol..

[31] Lehmann B., Meurer M. (2010). Vitamin D metabolism. Dermatol. Ther..

[32] Kato S. (2000). The function of vitamin D receptor in vitamin D action. J. Biochem..

[33] Jones G., Strugnell S.A., DeLuca H.F. (1998). Current understanding of the molecular actions of vitamin D. Physiol. Rev..

[34] Deeb K.K., Trump D.L., Johnson C.S. (2007). Vitamin D signalling pathways in cancer: Potential for anticancer therapeutics. Nat. Rev. Cancer.

[35] Gombart A.F., Luong Q.T., Koeffler H.P. (2006). Vitamin D compounds: Activity against microbes and cancer. Anticancer Res..

[36] O’Kelly J., Morosetti R., Koeffler H.P. (2005). Vitamin D and hematological malignancy. Vitamin D.

[37] Krishnan A.V., Feldman D. (2011). Mechanisms of the anti-cancer and anti-inflammatory actions of vitamin D. Annu. Rev. Pharmacol. Toxicol..

[38] Allavena P., Garlanda C., Borrello M.G., Sica A., Mantovani A. (2008). Pathways connecting inflammation and cancer. Curr. Opin. Genet. Dev..

[39] Mantovani A., Allavena P., Sica A., Balkwill F. (2008). Cancer-related inflammation. Nature.

[40] Bogh M.K. (2012). Vitamin D production after UVB: Aspects of UV-related and personal factors. Scand. J. Clin. Lab. Investig. Suppl..

[41] Hussein M.R. (2005). Ultraviolet radiation and skin cancer: Molecular mechanisms. J. Cutan. Pathol..

[42] Kammeyer A., Luiten R.M. (2015). Oxidation events and skin aging. Ageing Res. Rev..

[43] Karampinis E., Aloizou A.M., Zafiriou E., Bargiota A., Skaperda Z., Kouretas D., Roussaki-Schulze A.V. (2023). Non-Melanoma Skin Cancer and Vitamin D: The “Lost Sunlight” Paradox and the Oxidative Stress Explanation. Antioxidants.

[44] Vornicescu C., Ungureanu L., Șenilă S.C., Vesa Ș.C., Cosgarea R., Baican C.I., Mihu M.C. (2020). Assessment of sun-related behavior and serum vitamin D in basal cell carcinoma: Preliminary results. Exp. Ther. Med..

[45] Soares A.M., Szejnfeld V.L., Enokihara M.Y., Michalany N., Castro C.H. (2018). High serum 25-hydroxyvitamin D concentration in patients with a recent diagnosis of non-melanoma skin cancer: A case-control study. Eur. J. Dermatol..

[46] Nejentsev S., Godfrey L., Snook H., Rance H., Nutland S., Walker N.M., Lam A.C., Guja C., Ionescu-Tirgoviste C., Undlien D.E. (2004). Comparative high-resolution analysis of linkage disequilibrium and tag single nucleotide polymorphisms between populations in the vitamin D receptor gene. Hum. Mol. Genet..

[47] Haussler M.R., Whitfield G.K., Haussler C.A., Hsieh J.C., Thompson P.D., Selznick S.H., Dominguez C.E., Jurutka P.W. (1998). The nuclear vitamin D receptor: Biological and molecular regulatory properties revealed. J. Bone Miner. Res..

[48] Zmuda J.M., Cauley J.A., Ferrell R.E. (2000). Molecular epidemiology of vitamin D receptor gene variants. Epidemiol. Rev..

[49] Uitterlinden A.G., Fang Y., Van Meurs J.B., Pols H.A., Van Leeuwen J.P. (2004). Genetics and biology of vitamin D receptor polymorphisms. Gene.

[50] Carling T., Rastad J., Akerström G., Westin G. (1998). Vitamin D receptor (VDR) and parathyroid hormone messenger ribonucleic acid levels correspond to polymorphic VDR alleles in human parathyroid tumors. J. Clin. Endocrinol. Metab..

[51] Arai H., Miyamoto K.I., Yoshida M., Yamamoto H., Taketani Y., Morita K., Kubota M., Yoshida S., Ikeda M., Watabe F. (2001). The polymorphism in the caudal-related homeodomain protein Cdx-2 binding element in the human vitamin D receptor gene. J. Bone Miner. Res..

[52] Seretis K., Bounas N., Papaspyrou F. (2023). Antibiotic Prophylaxis in Reduction Mammaplasty: A Network Meta-Analysis. Aesthet. Plast. Surg..

[53] Seretis K., Bounas N. (2023). The efficacy of different nerve blocks on postoperative pain and sequela in patients undergoing abdominoplasty: A network meta-analysis. Aesthet. Surg. J..

[54] Gates M., Gates A., Pieper D., Fernandes R.M., Tricco A.C., Moher D., Brennan S.E., Li T., Pollock M., Lunny C. (2022). Reporting guideline for overviews of reviews of healthcare interventions: Development of the PRIOR statement. BMJ.

