# Supplementary material for: The Association of Vitamin D with Non-Melanoma Skin Cancer Risk: An Umbrella Review of Systematic Reviews and Meta-Analyses
Source: Medicina (Kaunas). 2023 Dec 7;59(12):2130. doi: 10.3390/medicina59122130 (PMC10744959; doi:10.3390/medicina59122130)
Supplement: Supplementary file 1 [file medicina-59-02130-s001.zip › Table S2.pdf]

Table S2. Citation matrix of RCTs

Studies→ (SRs)<br>↓<br>(RCTs)	Abdelwahab 2022	Sutedja 2022	Caini 2021	Caini 2014
**Passarelli 2020**		✓	✓	
**Rosenberg 2016**		✓		
**Brinkhuisen 2016**		✓		
**Tang 2011**	✓		✓	✓
